# Reduced native T_1_ on cardiac magnetic resonance imaging as a novel marker of myocardial involvement in Niemann-Pick disease type B

**DOI:** 10.1016/j.ahjo.2025.100636

**Published:** 2025-10-10

**Authors:** Betim Redzepi, Panagiotis Antiochos, Christel Tran, Joana Vieira Barbosa, Ambra Masi, Meng Zhang, Revi Adheriyani, Belinda Campos-Xavier, Juerg Schwitter

**Affiliations:** a Cardiovascular Department of Cardiology, Division of Cardiology, Lausanne University Hospital (CHUV), Lausanne, Switzerland; b Cardiac MR Center of the University Hospital Lausanne (CHUV), Lausanne, Switzerland; c Division of Genetic Medicine, Lausanne University Hospital (CHUV), Lausanne, Switzerland; d Faculty of Biology and Medicine, University of Lausanne, UniL, Lausanne, Switzerland; e Division of Gastroenterology and Hepatology, Lausanne University Hospital (CHUV), Lausanne, Switzerland

**Keywords:** Niemann-Pick disease type B, Myocardial involvement, Cardiac magnetic resonance imaging, T_1_ mapping

## Abstract

**Background:**

Niemann-Pick disease type B (NPD-B) is a rare lysosomal storage disorder caused by biallelic mutations in the *SMPD1* gene, leading to deficient acid sphingomyelinase activity and lipid accumulation in various organs. Although cardiac involvement is known in similar disorders like Anderson-Fabry disease, the myocardial impact in NPD-B remains poorly characterized.

**Methods:**

Two adult patients with genetically confirmed NPD-B underwent multiparametric cardiac magnetic resonance (CMR) including native T1 mapping and late gadolinium enhancement (LGE). Clinical, biochemical, and histopathological data were reviewed.

**Results:**

Patient 1, a 59-year-old man with a homozygous *SMPD1* mutation (c.[Arg610del]), showed advanced pulmonary disease and liver steatosis. CMR revealed biventricular dilation with preserved systolic function and reduced native T1 values without LGE, suggesting myocardial lipid accumulation. Despite enzyme replacement therapy, the patient died of pulmonary complications 22 months later.

Patient 2, a 31-year-old woman with compound heterozygous *SMPD1* mutations (c.[739G > A], c.[1801G > A]), had cirrhosis, dyslipidemia, and stable lung disease. CMR showed normal T1 values and no cardiac abnormalities.

**Discussion/conclusions:**

This study compares two adult NPD-B patients who underwent CMR imaging. The first patient who died during follow-up showed reduced native T1 values, suggesting myocardial lipid accumulation, while the second had normal values and no complications during follow-up. These two patients are reported here to stimulate research in this field, as native T1 may aid in assessment of myocardial involvement. The two cases also suggest a potential influence of genetic mutations on disease severity. Given the risk of cardiac involvement in NPD-B, CMR — especially T1 mapping — may be a valuable tool for assessing myocardial infiltration. Future studies are needed to explore its correlation with genotype, disease progression, and treatment response.

## 1 Introduction

Niemann-Pick disease (NPD; OMIM #257200), also named acid sphingomyelinase deficiency, is a rare autosomal recessive disease caused by deficient acid sphingomyelinase activity. This deficiency results in sphingomyelin and other lipids accumulation within cells of the monocyte-macrophage system. This disorder exhibits significant variability in clinical presentation, age of onset, and severity across its subtypes. NPD types A (NPD-A) and B (NPD-B) result from biallelic pathogenic variants in the SMPD1 gene (chromosome 11p15.1–11p15.4), which impair acid-sphingomyelinase activity. Over 180 SMPD1 variants have been identified, contributing to the broad spectrum of clinical phenotypes [1]. NPD-B, or the chronic visceral type, is less severe due to residual enzyme activity and typically presents later in childhood. Clinical features include hepatosplenomegaly, progressive pulmonary infiltration, and lipid abnormalities contributing to atherosclerosis and an increased risk of myocardial infarction. However, the impact of these lipid abnormalities on cardiac disease is not well understood, and the contribution of myocardial lipid accumulation has not been thoroughly investigated.

We herein describe the first 2 documented adult NPD-B cases with cardiac magnetic resonance (CMR) tissue characterization, with the aim to stimulate research in this field to explore a potential role of CMR, particularly T_1_ mapping, as a biomarker of myocardial involvement.

## 2 Methods

We conducted a detailed clinical, genetic, and cardiac imaging evaluation of two adult patients with a confirmed diagnosis of Niemann-Pick disease type B (NPD-B), caused by biallelic mutations in the *SMPD1* gene. Genetic testing was performed using targeted sequencing to identify pathogenic or likely pathogenic variants. Liver biopsies were obtained in both patients to assess hepatic involvement and characterize the pattern of lipid accumulation.

Cardiac assessment was performed using transthoracic echocardiography and multiparametric CMR imaging on a Siemens Sola 1.5 Tesla scanner. The CMR protocol included cine imaging for cardiac morphology and function, native T1 mapping using a modified Look-Locker inversion recovery (MOLLI) sequence, and late gadolinium enhancement (LGE) for myocardial tissue characterization. Native T1 values were analyzed in the interventricular septum and lateral wall, and blood-corrected T1 values were calculated to account for hematocrit-dependent variability. Reference values were established from healthy controls scanned under identical technical conditions in our institution.

Biochemical analyses, including lipid profiles, were performed to evaluate systemic lipid abnormalities. Clinical progression, including respiratory function and hepatic pathology, was documented over time. Enzyme replacement therapy status and disease outcomes were recorded.

## 3 Results

### 3.1 Patient 1

**Fig. 1:**
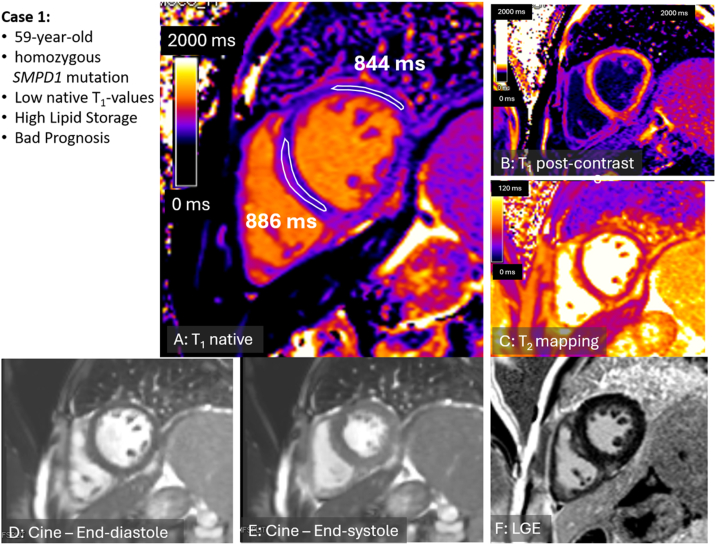
**Pre-contrast T1 mapping of patient 1.** Patient 1 died 22 months after the CMR study. A: Pre-contrast T_1_ mapping in patient 1 (with homozygous mutation) reveals decreased native-T_1_ relaxation times ranging from 886 to 844 ms in the septal and lateral wall, respectively; normal range at 1.5 T: 943–1073 ms [2]; blood-corrected native-T_1_ 910-866ms, normal value >939 ms [3]. B. post-contrast T_1_ mapping; C: T_2_-mapping revealed normal values; D and E: cine end-diastolic and end-systolic cine images, respectively; F: late‑gadolinium enhancement acquisition excludes macroscopic scar formation except subtle non-specific septal and inferior insertion point fibrosis.

A 59-year-old man with NPD-B caused by a homozygous mutation in the SMPD1 gene (c.[Arg610del]) was evaluated due to worsening dyspnea. The enzymatic activity was decreased. Liver biopsy showed microvesicular steatosis and enlarged macrophages with a foamy, vacuolated cytoplasm indicating abnormal lipid metabolism or storage. He also developed progressive interstitial lung disease, leading to a severe hypoxemic restrictive syndrome, that has required oxygen therapy over the last years. The NT-proBNP level was 537 ng/L. CMR (Siemens Sola, 1.5 T) showed dilation of both, left and right ventricles (left ventricular end-diastolic volume of 221 ml, indexed 131 ml/m2 and left ventricular mass of 127 g, indexed 75 g/m2) while maintaining normal systolic function (left ventricular ejection fraction of 55 %). Multiparametric CMR evaluation revealed decreased native-T_1_ values (886–844 ms in the septal and lateral wall, respectively; normal lower limit at 1.5 T: >943 ms [2], in our lab >935 ms; blood-corrected native-T_1_ 910-866ms, normal value >939 ms [3] (Fig. 1), suggesting possible myocardial lipid storage without specific LGE. Despite being treated with enzyme replacement therapy, the patient died from a pulmonary-related cause 22 months after the CMR examination.

### 3.2 Patient 2

**Fig. 2:**
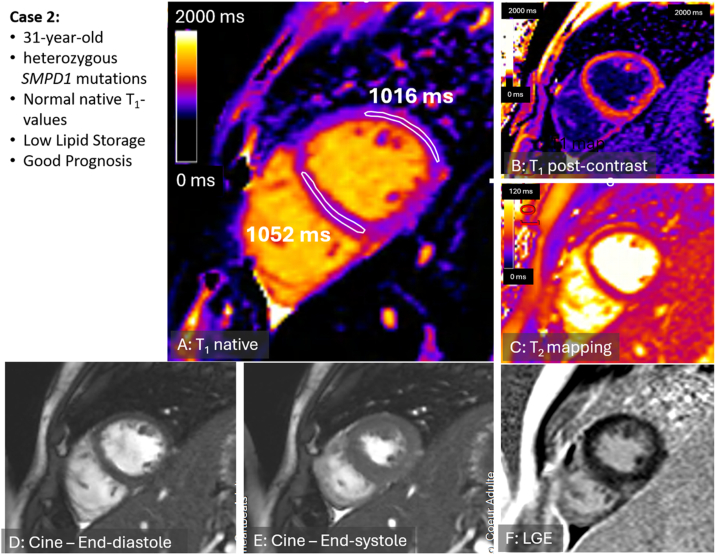
**Precontrast T1 mapping of patient 2.** Patient 2 showed a stable disease course over time. A. Pre-contrast T_1_ mapping in patient 2 (with compound heterozygous mutation) demonstrates normal native-T_1_ ranging from 1052 to 1016 ms in the septal and lateral wall, respectively; normal: 970–1116 ms [2]; blood-corrected native-T_1_ 1035-1000ms, normal value >939 ms [3]. B: post-contrast T_1_ mapping; C: T_2_-mapping revealed normal values; D and E: cine end-diastolic and end-systolic cine images, respectively; F: late‑gadolinium enhancement (LGE) acquisition excludes macroscopic scar formation except subtle inferior non-specific insertion point fibrosis.

A 31-year-old woman with NPD-B caused by a compound heterozygous mutation in the SMPD1 gene (c.[739G > A], pathogenic, and c.[1801G > A], likely pathogenic) was evaluated. The enzymatic activity was decreased. A liver biopsy confirmed the presence of advanced hepatic fibrosis, classified as cirrhosis, with lipid accumulation in hepatocytes and Kupffer cells, consistent with NPD-B. The patient also presented with diffuse interstitial lung disease with a stable course over time. Blood assessments revealed severe dyslipidemia: total cholesterol 8.4 mmol/L, LDL-cholesterol 5.3 mmol/l, HDL-cholesterol 0.3 mmol/l, and triglycerides 5.4 mmol/l. NT-proBNP level was within the normal range. Echocardiography showed normally sized, non-hypertrophied ventricles with preserved biventricular function (left ventricular ejection fraction of 55 %), which was confirmed by CMR (left ventricular end-diastolic volume of 148 ml, indexed 92 ml/m2 and left ventricular mass of 106 g, indexed 66 g/m2). A multiparametric evaluation revealed native-T_1_ values within the normal range (1052–1016 ms in the septal and lateral wall, respectively; normal: 970–1116 ms [2]; blood-corrected native-T_1_ 1035-1000ms, normal value >939 ms [3]) and no LGE, indicating no evidence of specific cardiac involvement (Fig. 2).

## 4 Discussion

**Fig. 3:**
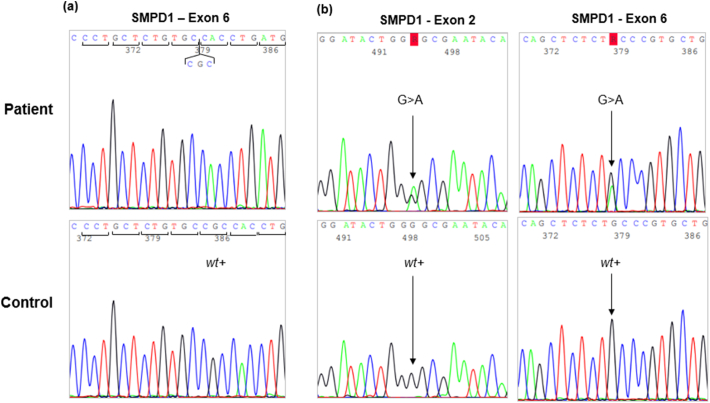
Partial electropherograms from the two patients. **(a)** Exon 6 sequence of the *SMPD1* gene from patient 1, in whom a homozygous in-frame deletion of three nucleotides (c. 1829_1831del, corresponding to the codon CGC) was identified (*upper row*) and a control (*lower row*). This deletion results in the loss of a single amino acid (p.Arg610del). **(b)** Sanger sequencing of compound heterozygous variants in the *SMPD1* gene from patient 2. Black arrows indicate the variants identified in exon 2 (c.739G>A; p.Gly247Ser) and exon 6 (c.1801G>A, p.Ala601Thr). The patient's sequence is shown in the *upper row*, compared to the normal control in the *lower row*.

In our study, the first patient demonstrated reduced native-T_1_ values potentially indicative of myocardial lipid storage, while the second patient exhibited normal values. Two possible explanations for this discrepancy include differences in genetic mutations and disease progression. The first patient harbors a homozygous SMPD1 mutation (c.[Arg610del]), while the second carries a compound heterozygous mutation consisting of one pathogenic variant and one likely pathogenic variant (c.[739G > A] and c.[1801G > A]). While no firm genotype-phenotype correlations have been established for this disease, our findings may suggest that certain genetic variants may be associated with differences in disease severity [4]. For case 1, the family history reveals that the patient has two sisters and one brother who are all in good health. Both of his parents are of Portuguese origin and are not consanguineous. His twin sister died at the age of four from a cardiac condition of unknown cause. For case 2, the patient has one sister, who died from NPD-B, and one brother who is also affected by NP-B. Both parents are carriers of the mutation. The father died due to heart failure secondary to cardiomyopathy. The first patient, a 59-year-old man, exhibits advanced disease features, including cirrhosis and severe pulmonary involvement, while the second patient was younger demonstrating earlier-stage pathology. The first patient died of interstitial pneumonia with progressive partial respiratory insufficiency, ongoing since 2015, while the second patient remains physically active without any functional limitations, is employed, and leads a normal family life. These findings underscore the importance of accounting for both, genetic and temporal factors when evaluating myocardial involvement in NPD-B (Fig. 3).

The pathophysiological mechanism of myocardial infiltration in NPD can be compared to Anderson-Fabry disease (AFD), another lysosomal storage disorder caused by mutations in the GLA gene, located on the X chromosome (Xq22.1). These mutations lead to alpha-galactosidase A deficiency resulting in the accumulation of glycosphingolipids in lysosomes of various cell types and widespread damage across multiple organ systems, including the cardiovascular system. In AFD, well established CMR findings include left ventricular hypertrophy with diffusely decreased myocardial native-T_1_ values that arise from myocardial lipid accumulation [3]. In the two cases, no endomyocardial biopsies (EMB) were obtained, which is certainly a relevant limitation. As the diagnosis was genetically confirmed in both cases, it was deemed not justified to perform EMBs.

Although recent guidelines recommend involving cardiologists in the evaluation of lipid profiles and coronary status in NPD, they do not specifically address follow-up for potential myocardial infiltration [5]. Given the potential for cardiac involvement in NPD-B, CMR imaging may be particularly useful in assessing these patients with respect to cardiac involvement and prognosis. Specifically, T_1_ mapping may provide insights into myocardial involvement, as lipid accumulation within the myocardium results in lower native T_1_-values. The features of the two patients described in this case report may stimulate research in this field to investigate the relation of myocardial native T_1_ values to demographics, disease severity/progression, enzymatic activity, and genotype. Such future studies would facilitate the understanding of the interaction between genetic factors and the disease and enable potential monitoring of the target organ in relation to the promising treatment recently introduced.

## 5 Conclusion

CMR findings of two adult patients with genetically confirmed NPD-B are reported to stimulate research in this field, as native T_1_ may aid in assessment of myocardial involvement. The first patient with homozygous SMPD1 mutation died during follow-up and showed reduced native T_1_ values, suggesting myocardial lipid accumulation, while the second patient with heterozygous SMPD1 mutations had normal native T_1_ values and no complications during follow-up. The two cases suggest that T_1_ mapping by CMR may detect myocardial lipid accumulation in NPD-B, thereby serving as a potential biomarker of disease severity. Genetic background and disease stage may influence cardiac involvement. Future studies are warranted to explore the role of CMR in monitoring myocardial pathology and guiding management in NPD-B.

## Statement of consent

Informed written consent has been obtained from the patients.

## CRediT authorship contribution statement

**Betim Redzepi:** Writing – review & editing, Writing – original draft, Validation, Software, Resources, Methodology, Investigation, Formal analysis, Conceptualization. **Panagiotis Antiochos:** Writing – review & editing, Writing – original draft, Validation, Software, Methodology, Investigation, Formal analysis, Conceptualization. **Christel Tran:** Writing – review & editing, Writing – original draft, Investigation, Formal analysis, Conceptualization. **Joana Vieira Barbosa:** Writing – review & editing, Writing – original draft, Investigation, Formal analysis. **Ambra Masi:** Writing – review & editing, Writing – original draft, Formal analysis. **Meng Zhang:** Writing – review & editing, Writing – original draft, Formal analysis. **Revi Adheriyani:** Writing – review & editing, Writing – original draft, Formal analysis. **Juerg Schwitter:** Writing – review & editing, Writing – original draft, Validation, Supervision, Methodology, Investigation, Formal analysis, Conceptualization.

## Ethical statement

This research was conducted in accordance with the ethical standards of the University Hospital of Lausanne (CHUV) and the principles outlined in the Declaration of Helsinki. Both participants provided informed consent prior to their involvement in the study. They were assured of the confidentiality of their responses and their right to withdraw at any time without consequence. All data were anonymized and securely stored in compliance with applicable data protection regulations.

## Sources of funding

The authors declare that no funds, grants, or other support were received for this article.

## Declaration of competing interest

The authors declare that they have no known competing financial interests or personal relationships that could have appeared to influence the work reported in this paper.

## Appendix A Supplementary data

Supplementary material 1 Image 1

Supplementary material 2 Image 2
