# Supplementary figures and images for: Reduced native T1 on cardiac magnetic resonance imaging as a novel marker of myocardial involvement in Niemann-Pick disease type B
Source: Am Heart J Plus. 2025 Oct 10;60:100636. doi: 10.1016/j.ahjo.2025.100636 (PMC12594934; doi:10.1016/j.ahjo.2025.100636)

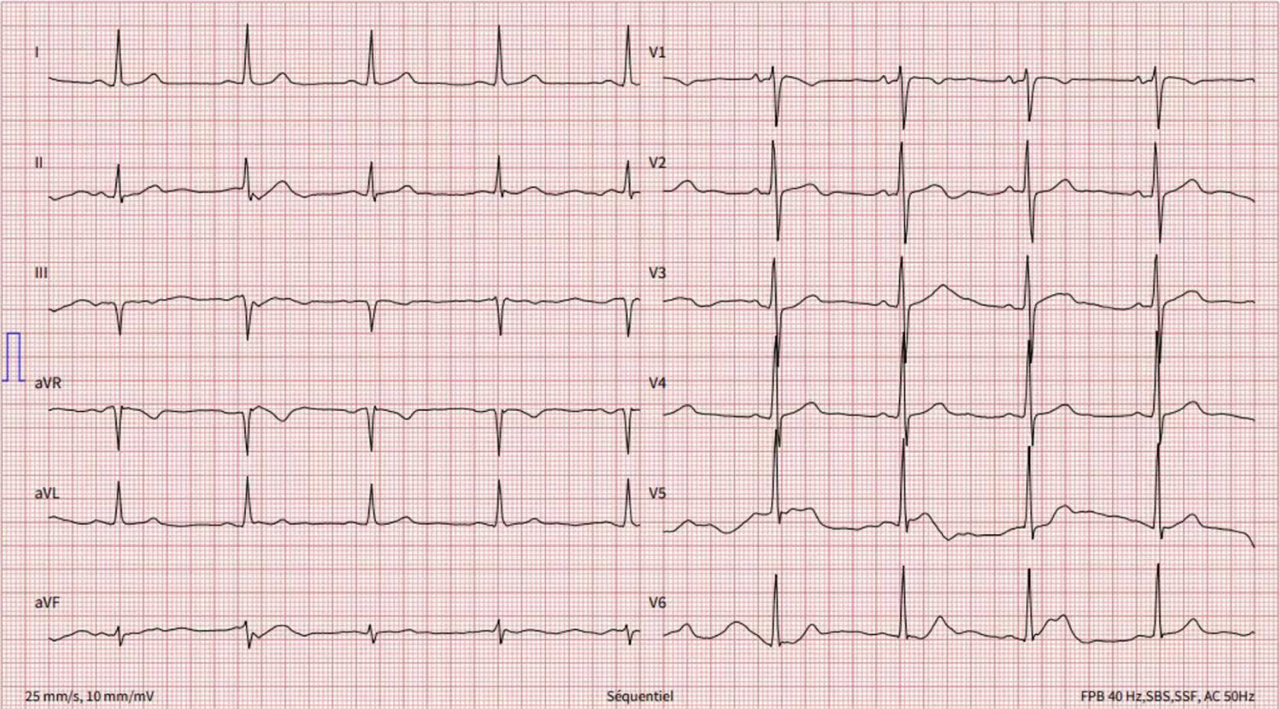

Supplement: Supplementary file 1 — Supplementary material 1 [file mmc1.zip › ECG_Case1.tif]

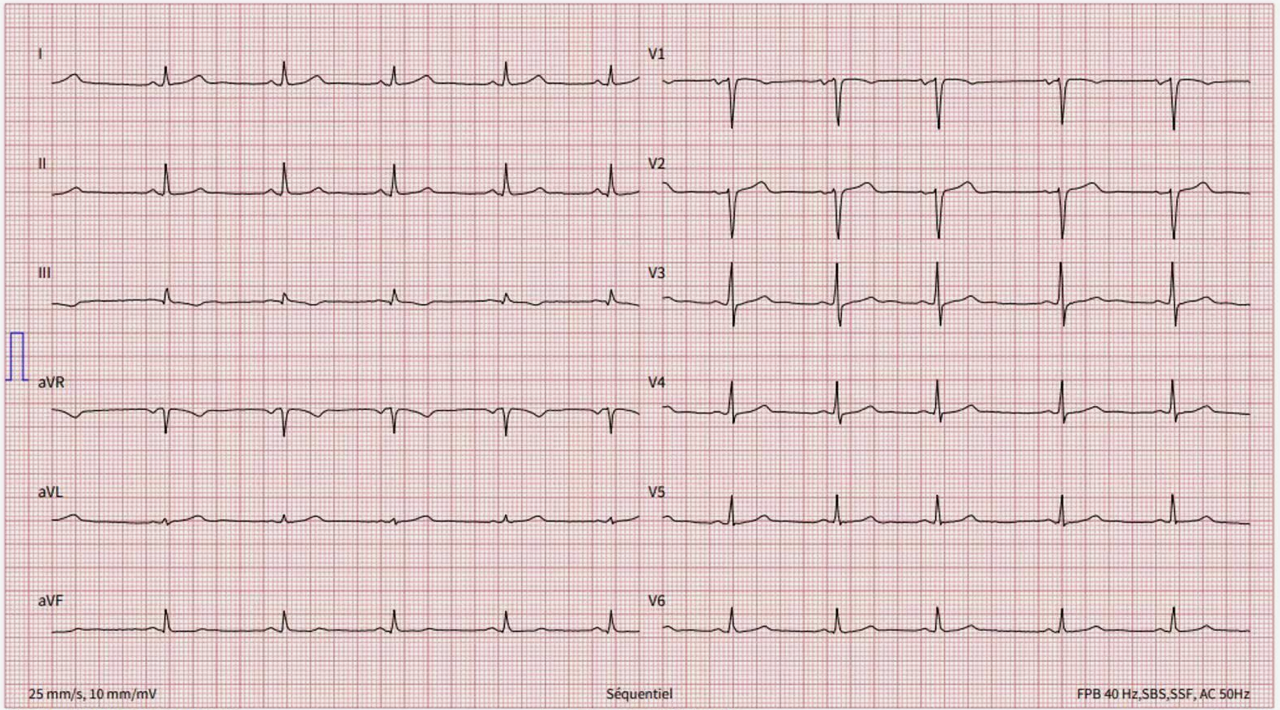

Supplement: Supplementary file 1 — Supplementary material 1 [file mmc1.zip › ECG_Case2.tif]
